# Natural product agonists of peroxisome proliferator-activated receptor gamma (PPARγ): a review

**DOI:** 10.1016/j.bcp.2014.07.018

**Published:** 2014-11-01

**Authors:** Limei Wang, Birgit Waltenberger, Eva-Maria Pferschy-Wenzig, Martina Blunder, Xin Liu, Clemens Malainer, Tina Blazevic, Stefan Schwaiger, Judith M. Rollinger, Elke H. Heiss, Daniela Schuster, Brigitte Kopp, Rudolf Bauer, Hermann Stuppner, Verena M. Dirsch, Atanas G. Atanasov

**Affiliations:** a Department of Pharmacognosy, University of Vienna, Austria; b Institute of Pharmacy/Pharmacognosy and Center for Molecular Biosciences Innsbruck (CMBI), University of Innsbruck, Austria; c Institute of Pharmaceutical Sciences, Department of Pharmacognosy, University of Graz, Austria; d Institute of Pharmacy/Pharmaceutical Chemistry and Center for Molecular Biosciences Innsbruck (CMBI), University of Innsbruck, Austria

**Keywords:** PPAR gamma, Nuclear receptor, Natural product, Nutrition, Diabetes, 9-(*S*)-HODE, (9*S*,10*E*,12*Z*)-9-hydroxyoctadeca-10,12-dienoic acid, AF-2, activation function-2, CAP, c-Cbl-associated protein, Cdk5, cyclin-dependent kinase 5, DCM, dichloromethane, DIO, diet-induced obesity, DPP-4, dipeptidylpeptidase 4, EMA, European Medicines Agency, FDA, Food and Drug Administration, Glut4, glucose transporter type 4, HDL, high-density lipoprotein, HUVEC, human umbilical vein endothelial cells, LBD, ligand-binding domain, LDL, low-density lipoprotein, MAPK, mitogen-activated protein kinase, MeOH, methanol, NF-κB, nuclear factor-kappaB, PPAR, peroxisome proliferator-activated receptor, RXR, retinoid X receptor, PDB, protein data bank, PPRE, peroxisome proliferator response element, SPPARMs, selective PPARγ modulators, TCM, traditional Chinese medicine, TNF-α, tumor necrosis factor alpha, Pioglitazone (PubChem CID: 4829), Magnolol (PubChem CID: 72300), Honokiol (PubChem CID: 72303), Falcarindiol (PubChem CID: 5281148), Resveratrol (PubChem CID: 445154), Amorfrutin 1 (PubChem CID: 10132170), Rosiglitazone (PubChem CID: 77999), Quercetin (PubChem CID: 5280343), (−)-Catechin (PubChem CID: 73160), Linolenic acid (PubChem CID: 5280934)

## Abstract

Agonists of the nuclear receptor PPARγ are therapeutically used to combat hyperglycaemia associated with the metabolic syndrome and type 2 diabetes. In spite of being effective in normalization of blood glucose levels, the currently used PPARγ agonists from the thiazolidinedione type have serious side effects, making the discovery of novel ligands highly relevant.

Natural products have proven historically to be a promising pool of structures for drug discovery, and a significant research effort has recently been undertaken to explore the PPARγ-activating potential of a wide range of natural products originating from traditionally used medicinal plants or dietary sources. The majority of identified compounds are selective PPARγ modulators (SPPARMs), transactivating the expression of PPARγ-dependent reporter genes as partial agonists. Those natural PPARγ ligands have different binding modes to the receptor in comparison to the full thiazolidinedione agonists, and on some occasions activate in addition PPARα (e.g. genistein, biochanin A, sargaquinoic acid, sargahydroquinoic acid, resveratrol, amorphastilbol) or the PPARγ-dimer partner retinoid X receptor (RXR; e.g. the neolignans magnolol and honokiol). A number of *in vivo* studies suggest that some of the natural product activators of PPARγ (e.g. honokiol, amorfrutin 1, amorfrutin B, amorphastilbol) improve metabolic parameters in diabetic animal models, partly with reduced side effects in comparison to full thiazolidinedione agonists. The bioactivity pattern as well as the dietary use of several of the identified active compounds and plant extracts warrants future research regarding their therapeutic potential and the possibility to modulate PPARγ activation by dietary interventions or food supplements.

## Graphical abstract

**Figure:**
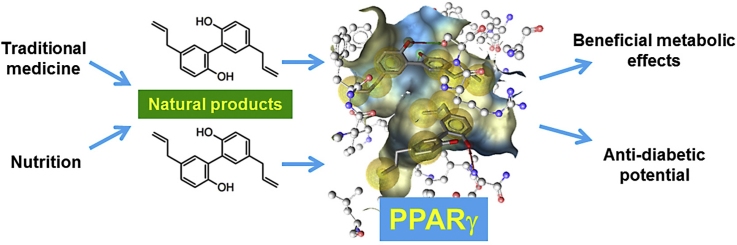

## 1 Significance of metabolic disorders

The metabolic syndrome is currently a major worldwide epidemic. It strongly associates with obesity, insulin resistance, type 2 diabetes, and cardiovascular diseases, which are major pathologies contributing to mortality and morbidity worldwide. At present the metabolic syndrome is already affecting more than a quarter of the world's adult population. Its prevalence is further growing in both adults and children due to a life style characterized by high calorie nutrition combined with low physical activity [1], [2].

The metabolic syndrome represents by definition a disorder related to imbalance of energy utilization and storage. Its features include abdominal obesity, hypertension, dyslipidemia (increased blood serum triglycerides; low high-density lipoprotein (HDL) and high low-density lipoprotein (LDL) cholesterol levels), insulin resistance with elevated fasting blood glucose, and glucose intolerance as well as establishment of pro-thrombotic and pro-inflammatory states [3]. People affected by the metabolic syndrome have a greater risk of developing cardiovascular diseases and type 2 diabetes. Moreover, recent research indicates that metabolic syndrome associated obesity causes chronic low-grade local tissue inflammation and increased susceptibility to other disease conditions such as fatty liver, sleep disturbances, cholesterol gallstones, polycystic ovary syndrome, asthma, and some types of cancer [3], [4].

The two main approaches in metabolic syndrome management are in the first place life style modifications that aim at restoring energy balance by reduced calorie intake and increased energy expenditure by physical activity, and on second place pharmaceutical interventions [1], [3]. Employed drugs target different relevant aspects of the metabolic syndrome such as body weight and fat distribution, insulin resistance, hypertension, dyslipidemia, hyperglycemia, or the established prothrombotic and proinflammatory state [3]. For the treatment of patients suffering from type 2 diabetes, aside from life-style alterations, insulin and insulin analogs were first applied [5]. Later a number of oral anti-hyperglycemic pharmaceuticals were developed and successfully used [6] including sulfonylureas (increasing insulin secretion) [7], biguanides (insulin sensitizers; e.g. metformin), alpha-glucosidase inhibitors (slowing the digestion of starch in the small intestine), meglitinides (increasing insulin secretion), dipeptidylpeptidase 4 (DPP-4) inhibitors (increasing insulin secretion) [6], as well as thiazolidinediones (agonists of PPARγ). Recent research strategies also explore targeting the nuclear factor-kappaB (NF-κB) pathway [8], mitogen-activated protein kinases (MAPK) signaling [9], fatty acid-binding proteins [10], as well as other targets involved in fatty acid metabolism [11], [12]. PPARγ, the molecular target of the thiazolidinediones, is particularly involved in the regulation of insulin sensitivity, inflammation, fatty acid storage, and glucose metabolism, and therefore represents an especially interesting pharmacological target which is able to simultaneously modulate several of the underlying pathologies of the metabolic syndrome [13], [14].

## 2 PPARγ and the metabolic regulation

PPARs belong to a subfamily of the nuclear receptor superfamily of ligand-inducible transcription factors [15]. To date, three PPAR isotypes encoded by separate genes have been identified, PPARα [16], PPARβ/δ, and PPARγ [17].

**Fig. 1:**
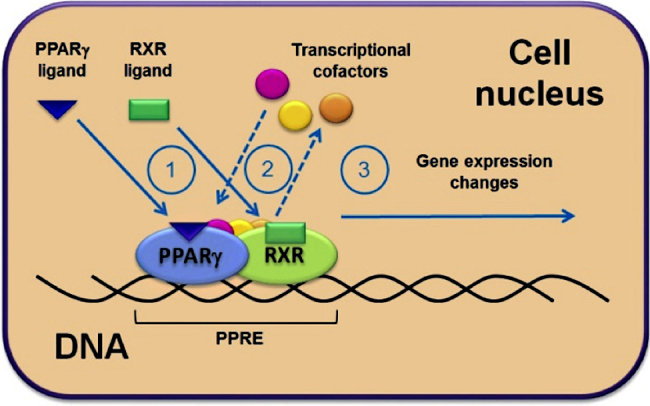
PPARγ transcriptional activation. (1) Binding of activating ligands to PPARγ and to its dimer partner RXR; (2) following the ligand binding there are conformational changes of the receptors, resulting in re-arrangement of the transcriptional complex and changes in the associated transcriptional cofactors; (3) resulting from this reorganization, the transcriptional complex is activated and initiates changes in the expression of the regulated PPARγ target genes.

PPARs mainly control the expression of gene networks involved in adipogenesis, lipid metabolism, inflammation, and the maintenance of metabolic homeostasis. As they can be activated by dietary fatty acids and their metabolites, they act as lipid sensors that, upon activation, are able to markedly redirect metabolism [18], [19], [20]. The gene transcription process is identical in all three PPAR subtypes (Fig. 1): After ligand binding, PPARs form heterodimers with another ligand-activated nuclear receptor, the retinoid X receptor (RXR). The PPAR-RXR heterodimer binds to peroxisome proliferator response elements (PPREs) in the promoter region of the respective target genes. The transcription process is then initiated upon recruitment of different transcriptional cofactors [21], [22], [23], [24] (Fig. 1).

The three PPAR isotypes possess a distinct tissue distribution and have different functions in the regulation of energy metabolism. PPARα is highly expressed in muscles, liver, heart, and kidney, and mainly regulates genes involved in the metabolism of lipids and lipoproteins [20], [25], [26], [27]. PPARβ/δ is abundantly expressed throughout the body but at low levels in the liver. It has emerged as an important regulator of lipid metabolism and energy balance primarily in adipose tissue, skeletal muscle, and the heart [25], [28], [29]. The PPARγ protein exists in two isoforms that are expressed from the same gene by utilizing distinct promoters and 5′exons. PPARγ2 differs from PPARγ1 by the presence of an additional stretch of 30 amino acid residues in the ligand-independent domain at the *N*-terminal end resulting in a higher transcriptional activity compared to PPARγ1 [30], [31], [32]. The two PPARγ isoforms also show a distinct expression pattern: PPARγ1 is abundantly expressed in adipose tissue, large intestine, and hematopoietic cells, and to a lower degree in kidney, liver, muscles, pancreas, and small intestine. PPARγ2 is restricted to white and brown adipose tissue under physiological conditions [25], [33], [34].

Endogenous ligands for PPARγ include fatty acids and prostanoids [19], [35] that act as weak agonists compared to the strong synthetic thiazolidinedione agonists [36], [37]. The question of whether PPARγ has some highly specific endogenous ligands or whether it operates as a rather promiscuous physiological lipid sensor activated in concert by a variety of fatty acids and eicosanoids is still not clearly resolved [38], [39], [40], [41], [42], [43].

In the human body, PPARγ is the master regulator of adipocyte differentiation, plays an important role in lipid metabolism and glucose homeostasis, modulates metabolism and inflammation in immune cells, as well as controls cell proliferation [44], [45], [46]. PPARγ is induced during the differentiation of preadipocytes into adipocytes [47], [48], [49]. The fact that PPARγ null mice are completely lacking adipose tissue clearly demonstrates that PPARγ is essential for adipocyte differentiation [50]. Furthermore, PPARγ directly activates many genes involved in adipocyte lipid storage [51], [52]. Adipose tissue is also the primary tissue responsible for the insulin-sensitizing effect of the thiazolidinedione-type PPARγ ligands. PPARγ controls the expression of numerous factors secreted from adipose tissue that influence insulin sensitivity positively (e.g. adiponectin, leptin) or negatively (e.g. resistin, tumor necrosis factor-α). In addition, PPARγ can directly modulate the expression of genes involved in glucose homeostasis, e.g. it upregulates glucose transporter type 4 (Glut4) and c-Cbl-associated protein (CAP) expression [53], [54]. PPARγ is also expressed in various immune system-related cell types, particularly in antigen-presenting cells such as macrophages and dendritic cells. In these cells, PPARγ does not only regulate genes related to lipid metabolism, but also immunity and inflammation related genes [55], [56], [57], [58]. Also the anti-atherosclerosis activity of PPARγ activating thiazolidinediones observed in animal models is thought to be generated primarily through modulation of PPARγ-regulated gene expression in macrophages [44], [59]. In addition to its metabolic and anti-inflammatory properties, PPARγ also modulates proliferation and apoptosis of many cancer cell types, and is expressed in many human tumors including lung, breast, colon, prostate, and bladder cancer. As natural and synthetic PPARγ activators have been found to inhibit cancer cell growth *in vitro* and in animal models, PPARγ might also be a target for new cancer therapies [44], [60], [61].

Aside from the availability of agonists and cofactors, the transcriptional activity of PPARγ is also regulated by its phosphorylation status, providing additional possibilities for fine-tuning [62], [63]. Phosphorylation of PPARγ at Ser273 by cyclin-dependent kinase 5 (Cdk5) was recently linked to obesity, and anti-diabetic PPARγ ligands (e.g. the thiazolidinedione rosiglitazone) were shown to inhibit the Cdk5-mediated phosphorylation of PPARγ in adipose tissue [62]. Moreover, several PPARγ ligands with poor agonistic activity but potent anti-diabetic effects *in vivo* revealed to be strong inhibitors of the PPARγ phosphorylation by Cdk5. The ligand's ability to suppress Ser273 phosphorylation correlated well with their anti-diabetic effectiveness but was independent of classical agonistic effects implied in some of the side-effects of PPARγ ligands currently used in clinics. Consequently, targeted inhibition of PPARγ Ser273 phosphorylation was suggested as a promising approach for development of a new generation of anti-diabetic agents [62].

While the application of PPARγ agonists is studied in many different disease conditions, the only approved use for PPARγ ligands so far is the application of thiazolidinediones (full PPARγ agonists) in type 2 diabetes. Thiazolidinediones first emerged as new class of drugs alleviating insulin resistance in patients with type 2 diabetes in the late 1990s [64], [65], [66]. The first approved drug of this class was troglitazone (CS-045), which became first available in March 1997 and was withdrawn from the US market in March 2000 [67]. Troglitazone activates preferentially PPARγ but is also a ligand of PPARα. As a drug counteracting type 2 diabetes, troglitazone increases insulin sensitivity and glucose tolerance in obese subjects [68], [69], [70], [71], [72], [73], [74], [75]. It was also demonstrated to inhibit the progression of early atherosclerotic lesions, to lower blood pressure, as well as to have favorable impact on other known cardiovascular risk factors [76], [77], [78]. In spite of its benefits in cardiovascular disease, troglitazone was removed from the market because it induced severe to fatal hepatotoxicity that outweighed its benefits for patients with diabetes [79], [80], [81], [82], [83], [84], [85].

Rosiglitazone (BRL-49653) and pioglitazone are both thiazolidinediones still in clinical use in many countries for glycemic control in the treatment of type 2 diabetes, although rosiglitazone-containing anti-diabetes medicines were taken off the market in the European Union following a European Medicines Agency (EMA) recommendation for suspension of the marketing authorizations (press release 23^rd^ of September 2010: EMA/585784/2010). In the United States the use of rosiglitazone was restricted by the Food and Drug Administration (FDA) in September 2010 and in November 2013 the restrictions were removed again, although according to the officially released FDA Drug Safety Communication (from 25^th^ of November 2013) “some scientific uncertainty about the cardiovascular safety of rosiglitazone medicines still remains”. Rosiglitazone has proven its effectiveness in reducing insulin resistance [86], [87], [88], [89], [90]. However, some meta-analyses indicated that among patients with impaired glucose tolerance or type 2 diabetes the use of rosiglitazone for at least 12 months was associated with a significantly increased risk of myocardial infarction and heart failure, as well as with an elevated risk of cardiovascular mortality [91], [92], [93], [94], [95]. Furthermore, some case reports rose concerns that the application of rosiglitazone might be associated with hepatocellular injury [96] and hepatic failure [97], side effects similar to those observed for troglitazone. Similar to rosiglitazone, treatment of type 2 diabetes patients with pioglitazone reduces insulin resistance significantly [98]. Compared to rosiglitazone, pioglitazone exerts beneficial effects on the plasma lipid profile, leading to a lower risk of acute myocardial infarction, stroke, or heart failure [99], [100], [101], [102], [103]. However, the clinical use of pioglitazone is also limited by the occurrence of several adverse events, including body-weight gain, fluid retention, and possibly bladder cancer [104], [105], [106].

## 3 PPARγ activation by natural products

The severe adverse effects of thiazolidinediones which led to their withdrawal from the market or restricted clinical application are suggested to be a result of full PPARγ activation, contrasting the weak agonistic effect of endogenous PPARγ ligands such as fatty acids and prostanoids [19], [107]. Therefore, great research efforts have recently been undertaken to explore the potential of selective PPARγ modulators (SPPARMs), compounds that improve glucose homeostasis but elicit reduced side effects due to partial PPARγ agonism based on selective receptor-cofactor interactions and target gene regulation [107], [108], [109]. An illustrative example for a recently identified SPPARM is *N*-acetylfarnesylcysteine, a compound with *in vitro* and *in vivo* effectiveness as both a full and partial agonist depending on the investigated PPARγ target gene [110]. A further research direction under consideration is to explore the therapeutic potential of dual- and pan-PPAR agonists activating simultaneously two or all three PPAR receptors, respectively [111], [112], [113], [114].

Medicinal plants have been used to treat various diseases for thousands of years, and since the 19^th^ century many bioactive pure compounds isolated from these plants became very successful drugs [115]. Moreover, still today natural products are an important source for the discovery and development of new drugs [116]. Natural products possess a high chemical scaffold diversity and are evolutionary optimized to serve different biological functions, conferring them a high drug-likeness and making them an excellent source for identification of new drug leads [117], [118], [119]. The traditional use of plant preparations can often give strong hints for the pharmacological effects of their ingredients. A study examining 119 clinically used plant-derived drugs found that 74% of them were indeed used for disease indications related to the traditional use of the medicinal plants from which the substances were isolated [120]. Not surprisingly, significant research efforts were undertaken to explore the PPARγ activating potential of a wide range of natural products originating from medicinal plants. Summarized in Table 1 are some of the most interesting examples of investigated sources, their use in traditional medicine, and the identified PPARγ-activating constituents. Noteworthy, along with plants and mushrooms applied in traditional medicines, PPARγ-ligands were often identified in plants that are common food sources, including the tea plant (*Camellia sinensis*), soybeans (*Glycine max*), palm oil (*Elaeis guineensis*), ginger (*Zingiber officinale*), grapes and wine (*Vitis vinifera*), and a number of culinary herbs and spices (e.g. *Origanum vulgare*, *Rosmarinus officinalis*, *Salvia officinalis*, *Thymus vulgaris*) (Table 1). The presence of PPARγ ligands in food products warrants an exploration whether this nuclear receptor may be effectively activated by the intake of nutraceuticals (by consumption of functional foods or by dietary supplements). Although most of the agonists identified in food sources are weak PPARγ agonists *per se*, the effects of their metabolites deserve further research to better estimate their preventive potential. While research in this direction is largely missing, a previous study reported that some main metabolites of flavonoid constituents from red clover (*Trifolium pratense*) have an up to 100-fold higher PPARγ binding affinity than their precursors [121].

**Table 1:** Species investigated as a source of PPARγ ligands, their traditional use, and identified activating natural products.

Species name	Traditional use	Identified PPARγ activating natural products
*Amorpha fruticosa* L. (Fabaceae)	Traditionally used to treat hypertension, hematomas, and contusions in China, Japan, and Korea [201]	Amorfrutins (in the fruits) [187]

*Astragalus membranaceus* Moench (Fabaceae)	In TCM used to reinforce qi and strengthen the superficial resistance, and promote the discharge of pus and the growth of new tissue [202]	Formononetin (in ethanolic extracts) [138]

*Bixa orellana* L. (Bixaceae)	In traditional medicine of India different parts of the plant are used as diuretic, laxative, antibilious, antiemetic and astringent agents, as blood purifier, in jaundice, in dysentery, and externally as scar-preventive [203]	Bixin and norbixin (in annatto extracts) [204]

*Camellia sinensis* (L.) Kuntze (Theaceae)	Used worldwide for the preparation of tea; used in the traditional medicine of India as stimulant, diuretic, and astringent. In China it is used in the treatment of diarrhea and dysentery [203]	(−)-Catechin (in green tea) [205]

*Cannabis sativa* L. (Cannabaceae)	In traditional medicine of India used as hallucinogenic, hypnotic, sedative, analgesic, and anti-inflammatory agent [203]	Δ9-Tetrahydrocannabinol [170]

*Chromolaena odorata* (L.) R.M. King & H. Rob. (Asteraceae)	In traditional medicine of Thailand used for the treatment of wounds, rashes, diabetes, and as insect repellent [206]	(9*S*,13*R*)-12-Oxo-phytodienoic acid (in chloroform-soluble extract from the whole plant) [207] and odoratin (in DCM extract) [208]

*Coix lacryma-jobi* var. *ma-yuen* (Rom. Caill.) Stapf ex Hook. f. (Poaceae)	In TCM used to invigorate the spleen function and promote urination, alleviate arthritis, arrest diarrhea, remove heat and facilitate the drainage of pus [202]	Hydroxy unsaturated fatty acids (in acetone extract from the seeds) [209]

*Commiphora mukul* (Hook. ex Stocks) Engl. (Burseraceae)	The oleo-gum-resin is used in traditional medicine of India for reducing obesity, as well as in the treatment of rheumatoid arthritis, osteoarthritis and sciatica [203]	Commipheric acid (in guggulipid, the ethyl acetate extract of the gum of the tree) [210]

*Cornus alternifolia* L.f. (Cornaceae)	Used in TCM as tonic, analgesic, and diuretic [211], [212]	Kaempferol-3-*O*-*β*-glucopyranoside (in 90% methanol extract from dried leaves) [211]

*Cymbopogon citratus* (DC.) Stapf (Poaceae)	In traditional medicine of India the leaves are used as stimulant, sudorific, antiperiodic, and anticatarrhal; the essential oil is used as carminative, depressant, analgesic, antipyretic, antibacterial, and antifungal agent [203]	Citral (in lemongrass oil) [213]

*Echinacea purpurea* (L.) Moench (Asteraceae)	Used in indigenous medicine of the native American Indians: external application for wounds, burns, and insect bites, chewing of roots for toothache and throat infections; internal application for pain, cough, stomach cramps and snake bites [214]	Alkamides (in *n*-hexane extract of the flowers) [215]

*Elaeis guineensis* Jacq. (Arecaceae)	In traditional African medicine different parts of the plant are used as laxative and diuretic, as a poison antidote, as a cure for gonorrhea, menorrhagia, and bronchitis, to treat headaches and rheumatism, to promote healing of fresh wounds and treat skin infections [216]	Tocotrienols (in palm oil) [217]

*Elephantopus scaber* L. (Asteraceae)	Different parts of the plant are used in traditional medicine of India as astringent agent, cardiac tonic, diuretic, to treat ulcers and eczema, in rheumatism, to reduce fever, and to eliminate bladder stones [203]	Deoxyelephantopin [218]

*Epimedium elatum* C. Morren & Decne. (Berberidaceae)	Used in TCM to reinforce the kidney yang, strengthen the tendons and bones, and relieve rheumatic conditions [202]	Acylated flavonol glycosides (in ethanol extract from the whole plant) [219]

*Euonymus alatus* (Thunb.) Siebold (Celastraceae)	Used in TCM to promote blood stasis to promote menstruation, remove toxic materials, subside swelling, and kill insects or parasites [202]	Kaempferol and quercetin [134]

*Glycine max* (L.) Merr. (Fabaceae)	The edible beans of the plant are used worldwide as a food and plant-based protein source [203]	Genistein (in soya beans) [135]

*Glycyrrhiza glabra* L. (Fabaceae)	Used in TCM to reinforce the function of the spleen and replenish qi, remove heat and counteract toxicity, dispel phlegm and relieve cough, alleviate spasmodic pain, and moderate drug actions [202]	5′-Formylglabridin, (2*R*,3*R*)-3,4′,7-trihydroxy-3′-prenylflavane, echinatin, (3*R*)-2′,3′,7-trihydroxy-4′- methoxyisoflavan, kanzonol X, kanzonol W, shinpterocarpin, licoflavanone A, glabrol, shinflavanone, gancaonin L, glabrone (in ethanol extract from the roots) [220]

*Glycyrrhiza foetida* Desf. (Fabaceae)	Used in the treatment of stomach and throat problems in traditional medicine of the Marrakech region in Morocco [221]	Amorfrutins (in the edible roots) [187]

*Glycyrrhiza inflata* Batalin (Fabaceae)	Used in TCM to reinforce the function of the spleen and replenish qi, remove heat and counteract toxicity, dispel phlegm and relieve cough, alleviate spasmodic pain, and moderate drug actions [202]	Licochalcone E (in roots) [222]

*Glycyrrhiza uralensis* Fisch. ex DC. (Fabaceae)	Used in TCM to reinforce the function of the spleen and replenish qi, remove heat and counteract toxicity, dispel phlegm and relieve cough, alleviate spasmodic pain, and moderate drug actions [202]	Flavonoids and 3-arylcoumarins (in ethanolic extract of the roots) [136]

*Limnocitrus littoralis* (Miq.) Swingle (Rutaceae)	In traditional Vietnamese medicine different parts of the plant have been used as an expectorant, antitussive product, for exudation, and the treatment of colds and fevers [223]	Meranzin (in ethyl alcohol/water (90/10, v/v) extract from the leaves) [224]

*Lycium chinense* Mill. (Solanaceae)	Used in TCM for the treatment of night-sweats, pneumonia, cough, hematemesis, inflammation, and diabetes mellitus [225]	Fatty acids (in root bark DCM extract) [128]

*Magnolia officinalis* Rehder & E.H. Wilson (Magnoliaceae)	Used in TCM to eliminate damp and phlegm, and relieve distension [202]	Magnolol [140], [193], [194] and honokiol [175], [190], [191], [192]

*Melampyrum pratense* L. (Orobanchaceae)	Used in traditional Austrian medicine for the treatment of gout and rheumatism [122], [129]	Lunularin and fatty acids (in aerial parts DCM and MeOH extracts) [129]

*Momordica charantia* L. (Cucurbitaceae)	In traditional medicine of India different parts of the plant are used to relieve diabetes, as stomachic, laxative, antibilious, emetic, and anthelmintic agent. Also used for the treatment of cough, respiratory diseases, skin diseases, wounds, ulcer, gout, and rheumatism [203]	Cucurbitane-type triterpene glycosides [226]

*Notopterygium incisum* C.T. Ting ex H.T. Chang (Apiaceae)	Used in TCM for the treatment of rheumatism, cold, and headache [227]	Polyacetylenes (in roots and rhizomes DCM extract) [228]

*Origanum vulgare* L. (Lamiaceae)	Used as a culinary herb worldwide; used in the traditional medicine of India as emmenagogue, antispasmodic, carminative, and expectorant [203]	Biochanin A (in dried leaves) [137]

*Panax ginseng* C.A. Mey. (Araliaceae)	Used in TCM to reinforce the vital energy, to remedy collapse and restore the normal pulse, benefit the spleen and lung, promote the production of body fluids, and anchor the mind [202]	Ginsenoside 20(*S*)-protopanaxatriol [229] and ginsenoside Rb_1_ (in ginseng roots) [230]

*Pinellia ternata* (Thunb.) Ten. ex Breitenb. (Araceae)	Used in TCM to remove damp and phlegm, relieve nausea and vomiting, and eliminate stuffiness in the chest and epigastrium [202]	Fatty acids (in different apolar extracts from the rhizomes) [130]

*Pistacia lentiscus* L. (var. Chia) (Anacardiaceae)	Uses of the resin in traditional medicine of India: as carminative, diuretic, stimulant, and astringent [203]	Oleanonic acid (in Chios mastic gum) [131]

*Pseudolarix amabilis* (J. Nelson) Rehder (published as *Pseudolarix kaempferi* Gordon) (Pinaceae)	Used in TCM as dermatologic antifungal remedy [231]	Pseudolaric acid B (in extracts of the root and trunk barks) [232]

*Pueraria thomsonii* Benth. (Fabaceae)	Used in TCM for the treatment of fever, acute dysentery, diarrhea, diabetes, and cardiovascular diseases [233]	Daidzein (in ethanolic extracts) [138]

*Robinia pseudoacacia* var. *umbraculifer* DC. (Fabaceae)	In traditional medicine of India different parts of *Robinia pseudoacacia* are used as laxative, antispasmodic, and diuretic [203]	Amorphastilbol (in seed extract) [234]

*Rosmarinus officinalis* L. (Lamiaceae)	Used as a culinary herb worldwide; in traditional medicine of India essential oil from flowers and leaves is used as anti-inflammatory agent, astringent, antiseptic, stomachic, carminative, and externally in circulatory disorders; flowering tops and leaves are used as carminative and diuretic [203]	Carnosic acid and carnosol (in ethanolic extract of rosemary) [235]

*Salvia officinalis* L. (Lamiaceae)	Used as a culinary herb worldwide; in traditional medicine of India different parts of the plant are used as astringent, anti-inflammatory, carminative, antispasmodic, antiseptic, hypoglycaemic, anti-asthmatic, cholagogue, emmenagogue, antisudoriferous, diaphoretic, and antipyretic agent, as well as for the treatment of sore throat, laryngitis, tonsillitis, and stomatitis [203]	Carnosic acid and carnosol (in ethanolic extract of sage) [235]; as well as 12-*O*-methyl carnosic acid and α-linolenic acid (in DCM extract of sage) [132]

*Sambucus nigra* L. (Adoxaceae)	In traditional medicine of India different parts of the plant are used as anti-inflammatory, anti-catarrhal, diuretic, and emetic agent, as well as for the treatment of common cold, influenza, nasal catarrh, and sinusitis [203]	α-Linolenic acid, linoleic acid, and naringenin (in MeOH extract of elderflowers) [133]

*Saururus chinensis* (Lour.) Baill. (Saururaceae)	In traditional Korean medicine aerial parts of the plant are used for the treatment of edema, jaundice, gonorrhea, and several inflammatory diseases [236]	Saurufuran A (in roots) [237]

*Silybum marianum* (L.) Gaertn. (Asteraceae)	Widely used worldwide as a supportive agent in the treatment of a variety of liver diseases; used in TCM to clear heat and relieve toxic material, to soothe the liver and to promote bile flow [202]	Isosilybin A (in silymarin, a phenolic mixture from the fruits of the plant) [238]

*Terminalia bellerica* Roxb. (Combretaceae)	The fruits are used in traditional medicine of India to treat anemia, asthma, cancer, diarrhea, hypertension, inflammation, and rheumatism [239]	Gallotannins (in the fruits) [240]

*Thymus vulgaris* L. (Lamiaceae)	Used as a culinary herb worldwide; used in traditional medicine of India as antiseptic, antibacterial, antifungal, antiviral, antispasmodic, mild sedative, and expectorant, for coughs and common cold [203]	Carvacrol (in thyme oil) [241]

*Trifolium pratense* L. (Fabaceae)	Used in traditional medicine of India as deobstruent, antispasmodic, expectorant, sedative, anti-inflammatory, and anti-dermatosis agent [203]	Isoflavones (in red clover extracts) [121]

*Vitis vinifera* L. (Vitaceae)	Widely used worldwide as food (grapes) and for beverage preparation (wine); used in traditional medicine of India in prescriptions for cough, respiratory tract catarrh, subacute cases of enlarged liver and spleen, as well as in alcohol-based tonics (Aasavs) [203]	Ellagic acid, epicatechin gallate, flavonoids (in grapes and wine) [242]

*Wolfiporia extensa* (Peck) Ginns (published as *Poria cocos* F.A. Wolf) (Polyporaceae)	In TCM this mushroom is used to cause urination, invigorate the spleen function, and calm the mind [202]	Dehydrotrametenolic acid (in dried sclerotia) [243]

*Zingiber officinale* Roscoe (Zingiberaceae)	Widely used as a spice worldwide; in TCM fresh rhizomes are used to dispel pathogenic factors from exterior and eliminate cold, arrest vomiting by warming the middle-energizer, remove phlegm and arrest cough; dried rhizomes are used to dispel cold from the spleen and the stomach, promote recovery from collapse, and warm the lung to expel retained morbid fluids [202]	6-Shogaol (in ginger roots) [244]

Although in some occasions the traditional use of the species presented in Table 1 might give hints for bioactivities linked to PPARγ activation, it is important to underline that the applications of traditional preparations often cover a broad range of symptoms that are unlikely to be related to PPARγ action (e.g. *Echinacea purpurea* is traditionally used for the treatment of wounds, burns, insect bites, toothache, throat infections, pain, cough, stomach cramps and snake bites; in this example the range of traditional uses is very likely linked to diverse bioactivities resulting from the interaction with different molecular targets).

While even many more plant extracts are reported to activate PPARγ [122], [123], [124], [125], [126], [127], Table 1 mainly summarizes studies that identified bioactive compounds present in the respective extracts. One reason for frequently omitting the identification of bioactive compounds might be the very high number of medicinal plant extracts inducing PPARγ activation in general. For example, a recent study examining the PPARγ transactivation potential of extracts from traditional Austrian medicinal plants identified that 40 out of 71 studied herbal drugs (56% hit rate) are able to induce PPARγ activation when tested at a concentration of 10 μg/mL [122]. This high number of active extracts makes it difficult to identify the bioactive compounds in each of them. In addition, the laborious phytochemical analysis is often not rewarded with the identification of interesting novel PPARγ ligands but with the re-isolation of some ubiquitous plant constituents activating the receptor such as fatty acids [128], [129], [130], [131], [132], [133] or flavonoids [121], [133], [134], [135], [136], [137], [138].

Besides testing of extracts and bio-guided approaches, virtual screening emerged as an effective strategy for the discovery of novel PPARγ ligands from natural sources. Rupp et al. used descriptor-based Gaussian process regression to search for PPARγ agonists based on a data set of 144 published PPARγ ligands [139]. A combination of prediction models and manual inspection of the hit list yielded 15 compounds, which were experimentally evaluated against PPARα and PPARγ activation. Eight compounds exhibited agonistic activity towards either of these receptors or both. The most active compound, a truxillic acid derivative, was a selective PPARγ agonist with an EC_50_ of 10 μM. Petersen et al. performed a pharmacophore-based virtual screening of a database containing over 57,000 traditional Chinese medicine constituents [131]. The ligand-based pharmacophore model consisted of one hydrogen bond acceptor and three hydrophobic features and was based on a set of 13 selective, partial PPARγ agonists. The virtual hit list contained 939 entries. Exemplarily, one virtual hit, present in *Pistacia lentiscus*, was experimentally investigated involving the testing of the *Pistacia* oleoresin extract and the bio-guided fractionation of the active extract. These efforts led to the discovery of oleanonic acid as a modestly active partial PPARγ agonist. Fakhrudin et al. discovered dieugenol, magnolol, and tetrahydrodieugenol as partial PPARγ agonists [140]. They used a structure-based pharmacophore model to screen natural compound databases. Among the highly ranked hits, several neolignans were isolated or synthesized and experimentally tested for their *in vitro* activity against PPARγ. Dieugenol, tetrahydrodieugenol, and magnolol with EC_50_ values in the low micromolar or submicromolar range also induced adipocyte differentiation in 3T3-L1 adipocytes. Lewis et al. used docking to select natural products for evaluation against PPARγ and in a mouse model for irritable bowel disease [141]. The top-ranked virtual hit from the docking, α-eleostearic acid, showed activity in the PPARγ binding assay, the cell-based reporter assay, and the *in vivo* mouse model for irritable bowel syndrome. Salam et al. screened a small in-house natural product library using a multi-step docking protocol [142]. They selected 29 hits from the 200 docked compounds for experimental analysis in a functional PPARγ activity assay. Six compounds, psi-baptigenin, hesperidin, apigenin, chrysin, biochanin A, and genistein, showed EC_50_s in the low micromolar range. Finally, Tanrikulu et al. used a structure-based pharmacophore model based on the common interactions of four PPARγ X-ray crystal structures in complex with different agonists [143]. They screened the Analyticon database, which contains natural products and their semi-synthetic derivatives. Their efforts led to the discovery of two α-santonin derivatives as PPARγ activators, while α-santonin itself was not active on the receptor. In summary, several 2D and 3D virtual screening approaches have successfully discovered structurally diverse natural product PPARγ activators, thereby indicating natural products as a rich source for novel PPARγ agonists.

A selection of natural products well characterized as PPARγ ligands is presented in Table 2. The PPARγ-agonistic effects of endogenous (e.g. fatty acids, prostanoids) [19], [26], [144], [145], [146], [147], [148], [149], [150], [151] and synthetic [13], [151], [152], [153] ligands of the receptor have been reviewed in numerous previous articles and therefore will not be discussed here. Natural products reported to activate or bind PPARγ with EC_50_ or respectively IC_50_ above 50 μM were considered as less relevant and were therefore omitted from Table 2. While numerous natural products were so far shown to interfere with PPARγ activity or expression (Table 1 and references [142], [154], [155], [156], [157], [158], [159], [160], [161], [162], [163], [164], [165], [166], [167], [168], [169], [170], [171], [172], [173]), the compounds depicted in Table 2 did not only show effectiveness in a cell model responsive to PPARγ activation (e.g. activation of PPARγ-dependent reporter gene expression), but also to directly bind to the receptor in an *in vitro* binding assay using purified PPARγ protein. While a binding assay with a purified receptor is one of the most direct approaches to confirm the potential of a compound to physically interact with PPARγ, application of a protein-based *in vitro* assay alone is not sufficient to assure that the respective compound can act also in intact cells (since the compound might not be able to reach PPARγ that is located inside the cell nucleus, due to various reasons such as inability to penetrate cellular membranes, extrusion from the cells mediated by membrane efflux transporters, metabolic transformation to products that do not bind PPARγ etc.). On the other side, the use of cellular models alone does not ensure that the studied compound is a direct receptor ligand, since PPARγ activation as observed in a luciferase reporter model might also be caused by indirect effects (e.g. increase in PPARγ protein expression, activation of the PPARγ dimer partner RXR). The 20 natural products covered in Table 2 include representatives of seven structural classes (flavonoids, neolignans, stilbenes, amorfrutins, polyacetylenes, sesquiterpene lactones, and diterpenequinone derivatives). This structural variety is consistent with the known ability of the PPARγ ligand-binding domain (LBD) to accommodate a diversity of chemical scaffolds due to the large size of the binding site cavity and its adaptability through the flexibility of side chains [43], [174]. With the exceptions of 6-hydroxydaidzein and (−)-catechin, all of the compounds reviewed in Table 2 revealed to be SPPARMs displaying partial agonistic effects towards PPARγ-dependent reporter gene expression. Genistein, biochanin A, sargaquinoic acid, sargahydroquinoic acid, resveratrol, and amorphastilbol were shown to be dual agonists able to activate also PPARα along with PPARγ (Table 2). Genistein also exerts estrogenic activity at low concentrations, leading to a concentration-dependent preferential activation of PPARγ or estrogen receptor, translating into opposite effects on osteogenesis and adipogenesis [135]. Six of the natural products, i.e. honokiol [175], magnolol [176], resveratrol [177], [178], [179], [180], [181], [182], [183], [184], [185], [186], amorfrutin 1 [187], amorfrutin B [188], and amorphastilbol [189], have been demonstrated to improve blood glucose levels and other relevant parameters in animal models of diabetes, on some occasions with reduced side effects in comparison to full thiazolidinedione PPARγ ligands (Table 2). In particular honokiol, amorfrutin 1, amorfrutin B, and amorphastilbol reduced weight gain in diabetic animal models. Furthermore, some of these compounds did not display adverse liver effects such as hepatomegaly (amorphastilbol) and hepatotoxicity (amorfrutin 1, amorfrutin B), and amorfrutin B also lacked adverse effects associated with osteoblastogenesis and fluid retention (Table 2). Among the studied natural products, amorfrutin 1 is the only one that was investigated so far for interference with PPARγ Ser273 phosphorylation and was found to suppress phosphorylation at this residue in the visceral white adipose tissue of diet-induced obesity (DIO) mice [187]. An interesting distinct mode of agonism is exerted by the neolignans honokiol and magnolol, which are dual agonists of PPARγ and its dimer activation partner RXR [140], [175], [190], [191], [192], [193], [194].

**Table 2:** Natural products activating PPARγ.

Bioactive compound	Notes
**Flavonoids**	
	Binds to purified human PPARγ with IC_50_ = 3.9 [127] or 7.2 μM [137], activates chimeric Gal4-PPARγ-dependent reporter gene expression as partial agonist (with EC_50_ = 15.6 μM and maximal efficacy around 3-fold lower than rosiglitazone) [195], and antagonizes the effect of rosiglitazone (1 μM) upon co-treatment (with IC_50_ = 21.8 μM) [195], antagonizes the adipogenesis inducing action of rosiglitazone (1 μM) in 3T3-L1 cells upon co-treatment (at 5–20 μM) [195], regulates several PPARγ-dependent genes as a weak partial agonist/antagonist, but acts as a full PPARγ agonist on GLUT4 expression in 3T3-L1 cells (at 10–20 μM) [195], counteracts (at 1–5 μM) the IL-8 secretion in human corneal epithelial cells exposed to hypertonic stress or to the PPARγ antagonist GW9662 (at 1 μM) [195], was co-crystallized with the PPARγ LBD whereby luteolin and myristic acid simultaneously bind to the LBD (PDB: 3sz1) [195]

	Binds to recombinant human PPARγ (IC_50_ reported to be 26.0 [134], 5.7 [242], or 2.8 μM [127]), activates PPARγ-dependent reporter gene expression as partial agonist when applied as a single treatment, and antagonizes the effect of rosiglitazone upon co-treatment (at 1–100 μM) [134], induces the insulin-dependent glucose uptake but not adipogenesis in 3T3-L1 cells (at 5–50 μM) [134], inhibits rosiglitazone-induced 3T3-L1 cell differentiation (at 5–50 μM) [134]

	Binds to recombinant human PPARγ (IC_50_ = 23.1 [134], 30 [242] or 49.9 μM [245]), activates PPARγ-dependent reporter gene expression as partial agonist when applied as a single treatment, and antagonizes the effect of rosiglitazone upon co-treatment (at 1–100 μM) [134], induces the insulin-dependent glucose uptake but not adipogenesis in 3T3-L1 cells (at 5–50 μM) [134], inhibits rosiglitazone-induced 3T3-L1 cell differentiation (at 5–50 μM) [134]

	Binds to purified PPARγ-LBD with IC_50_ = 9.9 μM [205], activates PPARγ-dependent reporter gene expression as full agonist with EC_50_ of around 2 μM [205], modulates expression of PPARγ target genes, and promotes adipocyte differentiation of human bone marrow mesenchymal stem cells [205]

	Binds to purified PPARγ (IC_50_ = 3.8 μM) and activates chimeric Gal4-PPARγ-dependent reporter gene expression as partial agonist (with EC_50_ = 3.8 μM and maximal efficacy around 3-fold lower than rosiglitazone) [127], [245]

	Binds to purified human PPARγ with IC_50_ = 19.6 [121] or 23.7 μM [137], activates chimeric Gal4-PPARγ-dependent reporter gene expression as partial agonist (with EC_50_ = 39.5 μM and maximal efficacy around 3-fold lower than pioglitazone) [121], induces adipogenesis in 3T3-L1 cells (at 1-5 μM) [138], activates PPARγ promoter activity in HUVEC transfected with PPRE-reporter plasmids and inhibits monocyte adhesion to TNF-α activated HUVEC in the presence of flow (at 1 μM) [246], activates also chimeric Gal4-PPARα-dependent reporter gene expression [138], [247]

	Binds to purified human PPARγ with *K*_*i*_ = 5.7 [135] or 22.5 μM [121], activates chimeric Gal4-PPARγ-dependent reporter gene expression as partial agonist (with EC_50_ = 18.7 μM and maximal efficacy around 4-fold lower than pioglitazone) [121], induces adipogenesis in 3T3-L1 cells (at 1-30 μM) [138], activates PPARγ promoter activity in HUVEC transfected with PPRE-reporter plasmids and inhibits monocyte adhesion to TNF-α activated HUVEC in the presence of flow (at 1 μM; the monocyte adhesion effect was abolished upon siRNA silencing of PPARγ) [246], activates also the transcriptional activity of PPARα [138], [247], [248], [249], was shown to act as an estrogen at low concentrations (≤1 μM) and as a ligand of PPARγ at high concentrations (>1 μM) leading to concentration-dependent opposite effects on osteogenesis and adipogenesis [135]

	Binds to purified human PPARγ with IC_50_ = 3.3 μM [121], activates chimeric Gal4-PPARγ-dependent reporter gene expression as full agonist with EC_50_ = 48.6 μM [121]

	Binds to purified human PPARγ with IC_50_ = 16.7 μM [121], activates chimeric Gal4-PPARγ-dependent reporter gene expression as partial agonist with EC_50_ = 27.7 μM and maximal efficacy around 5-fold lower than rosiglitazone [121]

**Neolignans**	
	Dual agonist of PPARγ and RXR [175], [190], [191], [192], binds to purified human PPARγ (*K*_*i*_ = 22.9 μM) [175], activates PPARγ-dependent reporter gene expression as partial agonist (EC_50_ = 3.9 μM) [175], induces glucose uptake but not adipogenesis in 3T3-L1 cells (at 1–10 μM) [175], decreases blood glucose levels in diabetic KKAy mice with simultaneous suppression of weight gain [175]

	Dual agonist of PPARγ and RXRα [140], [193], [194], binds to purified human PPARγ (*K*_*i*_ = 2.0 μM) [140], activates PPARγ-dependent reporter gene expression as partial agonist (EC_50_ = 1.6 μM) [140], induces the recruitment of TRAP220/DRIP-2 coactivator peptide to purified PPARγ (with EC_50_ of around 0.5 μM and maximal efficacy around 3-fold lower than pioglitazone) [140], induces adipogenesis [140], [194] and glucose uptake [194] in 3T3-L1 cells (at 10 μM), decreases fasting blood glucose and plasma insulin levels and prevents or retards diabetic nephropathy in type 2 diabetic Goto-Kakizaki rats [176], was co-crystallized with the RXRα-LBD (PDB: 3r5 m) and the PPARγ-LBD (PDB: 3r5n) [193]

**Stilbenes**	
	Binds to purified human PPARγ (*K*_*i*_ = 1.37 μM) [250], activates chimeric Gal4-PPARγ-dependent reporter gene expression as partial agonist (at 50–100 μM) [251], inhibits rosiglitazone-induced PPARγ luciferase reporter transactivation with IC_50_ = 27.4 μM [250], affects glucose and lipid metabolism as well as inflammation by interference with PPARγ in several *in vitro* and *in vivo* animal models [177], [178], [179], [180], [181], [182], [183], [184], [185] and improves insulin sensitivity in type 2 diabetic patients [186], is also a ligand of PPARα [250], [252], was co-crystallized with the PPARγ-LBD (PDB: 4jaz) [250]

	Binds to purified human PPARγ (IC_50_ = 0.85 μM) and activates human PPARγ-dependent luciferase reporter gene expression (EC_50_ = 5 μM; maximal fold activation of 83% as compared to the full agonist troglitazone) [234], binds and activates with a similar potency also PPARα [234], improves glucose and lipid impairment in db/db mice without significant side effects, such as weight gain or hepatomegaly [189]

**Amorfrutins**	
	Binds to purified PPARγ (*K*_*i*_ = 0.24 μM) and activates chimeric Gal4-PPARγ-dependent reporter gene expression as partial agonist (with EC_50_ = 0.46 μM and maximal efficacy 61% lower than rosiglitazone) [187], selectively modulates PPARγ gene expression networks in human adipocytes with a different pattern in comparison to synthetic PPARγ agonists [187], improves insulin resistance and other metabolic and inflammatory parameters without concomitant increase of fat storage or other unwanted side effects such as hepatotoxicity in diet-induced obese and db/db mice [187], blocks PPARγ Ser273 phosphorylation in DIO mice [187], was co-crystallized with the PPARγ-LBD (PDB: 2yfe) [187]

	Binds to purified PPARγ (*K*_*i*_ = 0.29 μM), and activates chimeric Gal4-PPARγ-dependent reporter gene expression as partial agonist (with EC_50_ = 1.2 μM and maximal efficacy 70% lower than rosiglitazone) [187], selectively modulates PPARγ gene expression networks in human adipocytes with a different pattern in comparison to synthetic PPARγ agonists [187], was co-crystallized with the PPARγ-LBD (PDB: 4a4v) [197]

	Binds to purified PPARγ (*K*_*i*_ = 0.019 μM) and activates chimeric Gal4-PPARγ-dependent reporter gene expression as partial agonist (with EC_50_ = 0.073 μM and maximal efficacy 4-fold lower than rosiglitazone) [188], induces partial recruitment of several PPARγ transcriptional coactivators [188], regulates gene expression in human adipocytes in a PPARγ-dependent manner [188], in insulin-resistant mice, it shows liver-protecting properties and improves insulin sensitivity, glucose tolerance, and blood lipid variables, without weight gain or adverse effects on osteoblastogenesis and fluid retention [188], was co-crystallized with the PPARγ-LBD (PDB: 4a4w) [197]

**Polyacetylenes**	
	Binds to purified human PPARγ (*K*_*i*_ = 3.1 μM) [228], activates PPARγ-dependent reporter gene expression as partial agonist (at 1–30 μM), and antagonizes the effect of rosiglitazone upon co-treatment [228], induces adipogenesis and glucose uptake in 3T3-L1 adipocytes at 10 μM [228]

**Sesquiterpene lactones**	
	Binds to purified PPARγ-LBD (*K*_D_ = 3.4 μM) but not to PPARα-LBD or PPARβ/δ-LBD [218], enhances the transcriptional activity of full-length PPARγ and Gal4-PPARγ-LBD chimera as a partial agonist (at 1–20 μM) [218], enhances the transcription activity of PPARγ upon co-treatment with non-saturating concentrations of rosiglitazone [218]

**Diterpenequinone derivatives**	
	Binds to purified PPARγ (IC_50_ = 0.255 μM) [253], activates PPARγ-dependent reporter gene expression as a partial agonist (at 1–30 μM) [253], enhances adipocyte differentiation in 3T3-L1 cells by increasing the expression of genes critical for adipocyte phenotype (at 10 μM) [253], activates also PPARα (at 1–30 μM) [253]

	Binds to purified PPARγ (IC_50_ = 0.725 μM) [253], activates PPARγ-dependent reporter gene expression as a partial agonist (at 1–30 μM) [253], enhances adipocyte differentiation in 3T3-L1 cells by increasing the expression of genes critical for adipocyte phenotype (at 10 μM) [253], activates also PPARα (at 1–30 μM) [253]

**Fig. 2:**
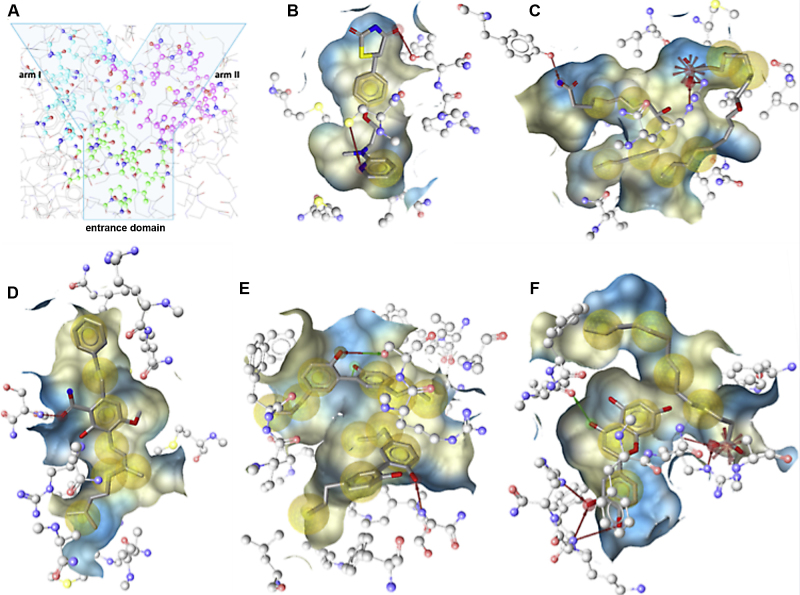
Binding modes of selected PPARγ ligands co-crystallized with PPARγ. (A) The Y-shaped PPARγ LBD composed of one entrance domain and two arms (arm I is substantially polar, arm II is mainly hydrophobic) [174]. Observed protein-ligand interactions are presented between the human PPARγ LBD and (B) the synthetic agonist rosiglitazone (PDB: 4ema), (C) the endogenous agonist 9-(*S*)-HODE binding as a homodimer (PDB: 2vsr), the natural ligands (D) amorfrutin B (PDB: 4a4w), (E) magnolol binding as homodimer (PDB: 3r5n), and (F) luteolin binding as a mixed dimer with myristic acid (PDB: 3sz1). The interactions were visualized by means of the software LigandScout [254] with the following color code: hydrogen bond acceptor (red arrow), hydrogen bond donor (green arrow), hydrophobic interaction (yellow sphere), and negative ionizable area (red star). The ligand binding pocket is depicted as surface; its colors are based on the lipo- and hydrophilicity. Contacts with active site water molecules are not shown.

Structural details for the binding to PPARγ LBD are revealed by receptor-ligand crystal structures solved for several natural products (Table 2 and Fig. 2). The PPARγ protein comprises an *N*-terminal regulatory domain, a central DNA-binding domain, and a *C*-terminal LBD (amino acids 204-477) [43], [195]. The LBD consists of 13 α-helices and a small four-stranded β-sheet [196]. Helix H12 of the ligand-dependent activation domain (activation function-2, AF-2) is essential for ligand binding and PPAR function. H12 and the loop between H2′ and H3 are the most mobile parts of the LBD. Ligand binding leads to a more rigid conformation of the LBD, which causes recruitment of coactivators and consequently transcription of target genes [197]. The PPARγ LBD is a large Y-shaped cavity that is composed of an entrance domain and two pockets, arm I and arm II (Fig. 2A) [198]. The large size and the flexibility of the binding pocket allow PPARγ to interact with structurally distinct ligands. No ligand is known that completely fills this large cavity [43]. However, it enables in some instances the simultaneous binding of two or even three molecules, which interact with the binding pocket as well as with each other, resulting in a more stable binding conformation [199]. Moreover, different ligands bind different areas in the PPARγ LBD, representing different binding modes. Depicted in Fig. 2 are the binding modes of a selection of ligands co-crystallized with the PPARγ LBD: the full thiazolidinedione agonist rosiglitazone (protein data bank (PDB) [200] entry PDB: 4ema [199], Fig. 2B); (9*S*,10*E*,12*Z*)-9-hydroxyoctadeca-10,12-dienoic acid (9-(*S*)-HODE) as a representative endogenous ligand that binds as a homodimer (PDB: 2vsr [43], Fig. 2C); the natural product amorfrutin B (PDB: 4a4w [197], Fig. 2D); the neolignan magnolol that binds as a homodimer (PDB: 3r5n [193], Fig. 2E); and the flavonoid luteolin binding concomitantly with myristic acid (PDB: 3sz1 [195], Fig. 2F).

In general, strong PPARγ agonists such as thiazolidinediones are known to bind to H12, whereas partial agonists stabilize the β-sheet and the H2′/H3 area. The full agonist rosiglitazone stabilizes H12 by building hydrogen bonds with Tyr473, which leads to coactivator recruitment [199]. Whereas just one molecule of the thiazolidinedione agonists such as rosiglitazone is binding to the LBD (PDB: 4ema [199], Fig. 2B), some endogenous ligands such as 9-(*S*)-HODE were demonstrated to activate the receptor as homodimers (PDB: 2vsr [43], Fig. 2C). The first 9-(*S*)-HODE molecule binds with its carboxy group via hydrogen bond to Tyr 473 of H12. This interaction is typical for carboxylate-containing ligands. The tail, which is located in an area also occupied by highly potent agonists, interacts *via* van der Waals contacts with Phe363 and other amino acids. The second molecule is located between H3 and the β-sheet, an area which is occupied also by synthetic partial agonists. Its carboxylate group forms a salt bridge with Arg288, an amino acid, which is not involved in the binding of thiazolidinediones [43].

The partial PPARγ agonists amorfrutin 1, 2, and B (PDB: 2yfe, PDB: 4a4 v, and PDB: 4a4w, respectively [187], [197]) are localized and oriented almost identically in the PPARγ LBD. They bind to and therefore stabilize the β-sheet as well as H3 of PPARγ by hydrogen bonds and van der Waals contacts. The reason for the high affinity of amorfrutins to PPARγ is the interaction of the carboxyl group to Ser342 of the β-sheet via hydrogen bonds. Also Arg288 of H3 is stabilized by amorfrutins. The replacement of Arg288 by threonins in PPARα and PPARβ/δ is likely the reason for the selective PPARγ activity of amorfrutins 1, 2, and B. However, there are also differences in their interactions with the LBD. Amorfrutin B shows significantly higher affinity than other reported amorfrutins, similar to that of rosiglitazone. This is caused by the long geranyl side chain, which forms additional hydrophobic interactions especially to Arg288 of H3 and to H4/5 [197].

According to the PDB: 3sz1, the PPARγ partial agonist luteolin binds to the PPARγ LBD simultaneously with the long-chain fatty acid myristic acid. The two molecules stabilize the β-sheet as well as the loop among H2′ and H3. Luteolin interacts *via* hydrogen bonds with Lys265 and His266 at the loop that links H2′ and H3 and builds hydrophobic contacts with various amino acids. Myristic acid occupies H3, H5, and H7 and interacts with Arg288 (H3) *via* a salt bridge. Luteolin and the carboxylate of myristic acid are connected *via* a water molecule through a hydrogen bond. This water molecule seems to be important for keeping luteolin in the LBD [195].

Similar to some endogenous ligands such as 9-(*S*)-HODE, two magnolol molecules were demonstrated to cooperatively occupy the PPARγ LBD. One magnolol molecule occupies AF-2, the other one the β-sheet. In AF-2, the hydroxyl group of magnolol makes a hydrogen bond with Ser289 in H3 and water-mediated hydrogen bonds with Tyr473. In the β-sheet, the hydroxyl group of the second magnolol forms a hydrogen bond with Ser342. Furthermore, there is also a water-mediated hydrogen bond in the β-sheet to magnolol. The magnolol structure is highly flexible due to the single bond connecting the two 5-ally-2-hydroxyphenyl moieties. It exhibits three different conformations when binding to PPARγ and RXRα, which bind two and one molecule of magnolol, respectively [193].

## 4 Concluding remarks

Natural products prove to be a rich source for the discovery of novel PPARγ ligands and many structurally diverse agonists of this receptor were recently identified from traditionally used medicinal plants or food sources. Interestingly, the majority of identified natural compounds are rather weak agonists of PPARγ, often activating the receptor as partial agonists, with activation pattern distinct from the full thiazolidinedione agonists and more similar to endogenous ligands with weaker activation potential such as fatty acids and prostanoids. Noteworthy, several PPARγ agonists were identified in plants used as culinary spices, beverages or food sources, opening the possibility to consider modulation of the activity of this nuclear receptor through dietary interventions. While most of the identified natural products only activate PPARγ as SPPARMs, some are dual agonists able to also activate PPARα (Table 2). An especially interesting activation pattern is observed for the neolignans magnolol and honokiol, which are ligands for both PPARγ and its dimer activation partner RXR. The neolignan honokiol and several other natural products have also demonstrated beneficial metabolic effects in diabetic animal models, with reduced side effects in comparison to full thiazolidinedione agonists. Many extracts from medicinal plants reported in the literature as PPARγ activators are so far not thoroughly investigated. The identification of their active constituents might provide further interesting ligands in the future.

In conclusion, a range of PPARγ activating natural products and plant extracts were recently described that bear a good potential to be further explored for therapeutic effectiveness as well as to be studied as potential dietary supplements to counteract the metabolic syndrome and type 2 diabetes.
